# Evaluating the implementation of community volunteer assessment and referral of sick babies: lessons learned from the Ghana Newhints home visits cluster randomized controlled trial

**DOI:** 10.1093/heapol/czu080

**Published:** 2014-09-11

**Authors:** Alexander Ansah Manu, Augustinus ten Asbroek, Seyi Soremekun, Thomas Gyan, Benedict Weobong, Charlotte Tawiah-Agyemang, Samuel Danso, Seeba Amenga-Etego, Seth Owusu-Agyei, Zelee Hill, Betty R Kirkwood

**Affiliations:** ^1^Kintampo Health Research Centre, Ghana Health Service, P.O. Box 200, Kintampo, Brong Ahafo Region, Ghana, ^2^Department of Public Health, Academic Medical Centre, Amsterdam, The Netherlands, ^3^Department of Nutrition and Public Health Intervention Research, London School of Hygiene and Tropical Medicine, Keppel Street, London WC1E 7HT, UK and ^4^Institute of Child Health, University College London, 30 Guilford Street, London WC1N 1EH, UK

**Keywords:** Assessment and referral, community, implementation, newborns

## Abstract

A World Health Organization (WHO)/United Nations Children's Fund (UNICEF) (2009) joint statement recommended home visits by community-based agents as a strategy to improve newborn survival, based on promising results from Asia. This article presents detailed evaluation of community volunteer assessment and referral implemented within the Ghana Newhints home visits cluster-Randomized Controlled Trial (RCT). It highlights the lessons learned to inform implementation/scale-up of this model in similar settings. The evaluation used a conceptual framework adopted for increasing access to care for sick newborns and involves three main steps, each with a specific goal and key requirements to achieving this. These steps are: sick newborns are identified within communities and referred; families comply with referrals and referred babies receive appropriate management at health facilities. Evaluation data included interviews with 4006 recently delivered mothers; records on 759 directly observed volunteer assessments and 52 validation of supervisors’ assessments; newborn care quality assessment in 86 health facilities and in-depth interviews (IDIs) with 55 mothers, 21 volunteers and 15 health professionals. Assessment accuracy of volunteers against supervisors and physician was assessed using Kappa (agreement coefficient). IDIs were analysed by generating and indexing into themes, and exploring relationships between themes and their contextual interpretations. This evaluation demonstrated that identifying, understanding and implementing the key requirements for success in each step of volunteer assessment and referrals was pivotal to success. In Newhints, volunteers (CBSVs) were trusted by families, their visits were acceptable and they engaged mothers/families in decisions, resulting in unprecedented 86% referral compliance and increased (55–77%) care seeking for sick newborns. Poor facility care quality, characterized by poor health worker attitudes, limited the mortality reduction. The important implication for future implementation of home visits in similar settings is that, with 100% specificity but 80% sensitivity of referral decisions, volunteers might miss some danger signs but if successful implementation must translate into mortality reductions, concurrent improvement in facility newborn care quality is imperative.

**KEY MESSAGES**

- In resource-constrained settings, community volunteers can be successfully used to identify through assessment and refer of sick newborn to health facilities as recommended in the WHO/UNICEF joint statement on home visits in 2009.
- Implementation of community volunteer assessment and referrals requires identification of key actions or strategies which should be monitored during implementation.
- The use of existing health systems structures such as district health management teams and beneficiary involvement in the planning allows for implementation at scale.
- Isolated community interventions will have limited impact unless coupled with concurrent improvement of quality within health facilities.

## Introduction

Improving access to care for sick newborns is key to reducing the 3.3 million babies who die each year within 28 days of birth (neonatal period) (Darmstadt *et al.* 2005; Oestergaard *et al.* 2011). The majority of these deaths occur in low- and middle-income countries (LMICs), in settings where most births and illness that lead to death occur at home, (Lawn *et al.* 2005; Kinney *et al.* 2010) with no health facility contacts (Lawn *et al.* 2005; Oestergaard *et al.* 2011). This is because families do not recognize newborn illness (Kumar *et al.* 2008; Syed *et al.* 2008; Choi *et al.* 2010) and when they do, care seeking is poor (Lawn *et al.* 2005; Awasthi *et al.* 2008; Bazzano *et al.* 2008; Kumar *et al.* 2008; Syed *et al.* 2008) and often besieged with barriers such as costs, distance, availability of services and social seclusion prohibiting out of home care seeking (Winch *et al.* 2005; Awasthi *et al.* 2008; Bazzano *et al.* 2008; Syed *et al.* 2008; Okyere *et al.* 2010). Community-based strategies are therefore urgently needed (Darmstadt *et al.* 2005).

The World Health Organization (WHO) and United Nations Children’s Fund (UNICEF) in 2009 issued a joint statement recommending home visits by community-based agents (CBAs) as a strategy to improve newborn survival (WHO/UNICEF 2009). This promotes examining babies in the first week after birth and referring any with danger signs or conditions requiring additional care, teaching families how to identify signs of illness and counselling on the importance of prompt health facility care seeking. This strategy was based on evidence from studies in Asia which successfully reduced neonatal mortality through home visits by community health workers (CHWs) (Bang *et al.* 2005; Baqui *et al.* 2008; Kumar *et al.* 2008; Azad *et al.* 2010; Darmstadt *et al.* 2010; Bhutta *et al.* 2011; Bhandari *et al.* 2012).

The Newhints cluster-randomized controlled trial (CRT) (Kirkwood *et al.* 2010) in Ghana is the first trial to evaluate this approach in sub-Saharan Africa. It demonstrated evidence of reduction in post-day 1 newborn mortality, achieved by increasing coverage of essential newborn care (ENC) practices and by improving access to care for sick newborns through high compliance with community volunteer referrals and improved care-seeking (Kirkwood *et al.* 2013; A Manu, G ten Asbroek, S Soremekun, B Weobong, T Gyan, C Tawiah-Agyemang, unpublished data). This article presents a detailed evaluation of the implementation of the assessment and referral component of the Newhints intervention and shares the lessons learned to inform scale-up and implementation of this core component in other settings.

## Methods

### Study setting and the Newhints Trial

#### Setting

Details of the Newhints intervention and the cluster randomized trial (CRT) are given elsewhere (Kirkwood *et al.* 2010). The trial was conducted in seven contiguous districts in the Brong-Ahafo region of Ghana covering 12 000 sqkm, (Kirkwood *et al.* 2010) a population of ∼700 000 (Ghana Health Service 2005) with over 120 000 women of reproductive age and more than 15 000 babies born each year. The neonatal mortality rate at baseline was 32/1000 livebirths (Kirkwood BR, Manu A, ten Asbroek AH, *et al.*, submitted for publication). Eighty per cent of the population live in villages comprising scattered compounds surrounded by farmlands and lacking modern infrastructure. The area is multi-ethnic, educational levels are low and subsistence farming is the main economic activity.

**Figure 1.**
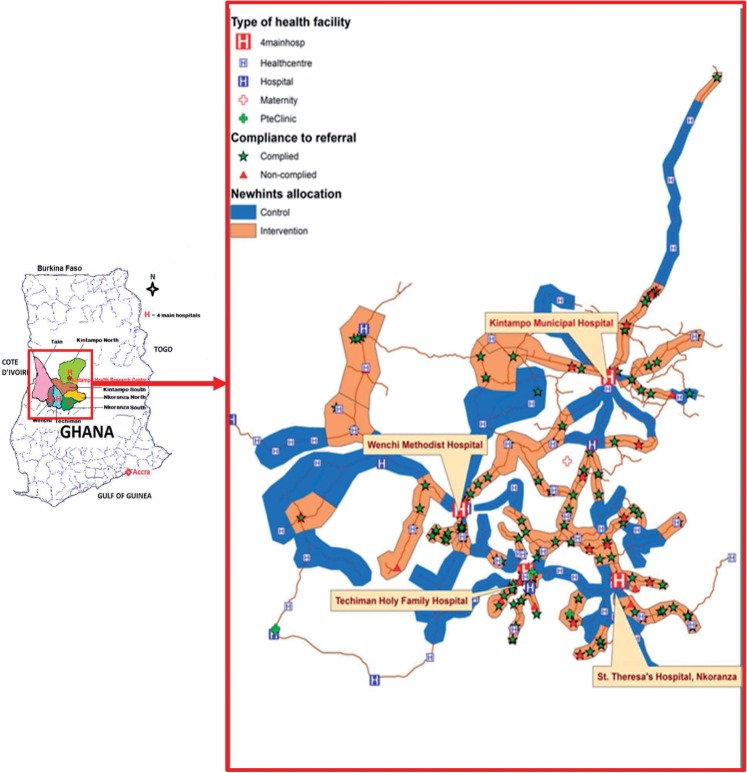
Map of the Ghana showing Newhints study districts and locations from where newborns were referred in Newhints.

Four main district hospitals located in urban centres (Figure 1) act as referral destinations for over 80 other facilities serving the area. All communities (populations of people living in a confined geographical area, either in villages or towns, but with the same chieftaincy or political administration) have community-based surveillance volunteers (CBSVs), selected by their communities to support district health management teams (DHMTs) in community mobilization for health programmes. They are predominantly male (∼80%) with at least primary education (>90%).

#### The Newhints’ cluster randomized trial

Newhints was an integrated intervention based on extensive formative research and developed in collaboration with DHMTs in the seven districts and input from national and international experts. The study area was divided into 98 Newhints supervisory zones, each of which comprised clusters of two to six contiguous villages or parts of a big town. The zones were so demarcated so that each had between 8 and 12 CBSVs. The CBSVs in 49 Newhints out of the 98 supervisory zones were trained to promote essential newborn care (ENC) practices through five home visits, two in pregnancy and three in the first week after birth, the time of the greatest vulnerability for the newborn (Lawn *et al.* 2005), to weigh and assess newborns for ten key danger signs (Table 1) and refer to health facilities when any was present (A Manu, G ten Asbroek, S Soremekun, B Weobong, T Gyan, C Tawiah-Agyemang, unpublished data). This simple checklist approach was adopted rather than an algorithm with branches and actions based on specific signs as this was both quicker to explain and more easily understood by community volunteers. CBSVs in the 49 control zones continued normal activities. The impact of the Newhints intervention was evaluated on the cohort of babies born between November 2008 and December 2009.

**Table 1.** Danger signs for referrals and coverage achieved

Assessment	Danger sign	Coverage of assessments	
		DOS (*N* = 759)	Process (*N* = 2795)
Ask			
How is the baby feeding?	1. Baby not breastfeeding well since birth or stopped breastfeeding	740 (97.5%)	
History of convulsion or fits since birth.	2. Baby convulsed or fitted since birth and not treated in a health facility	641 (84.5%)	
Check for			
Chest movements	3. Baby having lower chest in-drawing on inspiration	656 (86.4%)	
Palms and soles of the feet	4. Baby having yellow palms and soles	682 (89.9%)	
Lethargy/failure to move	5. Baby very weak and not moving at all or only moving when stimulated	671 (88.4%)	
Local infections	6. Baby having reddening around the ‘umbilicus’ or pus discharging from the stump, ‘skin pustules’ or purulent discharge from the eyes.	672 (88.5%)	
Measure			
Respiratory rate	7. Baby breathing too fast: 60 breaths or more per minute validated by a second count	742 (97.9%)	2662 (95.2%)
Temperature	8. Baby having fever: axillary temperature of 37.5°C or more	747 (98.4%)	2677 (95.8%)
	9. Baby too cold: axillary temperature of 35.4°C or less		
Weight	10. Less than 1.5 kg (red zone of the scale)	671 (88.4%)	2651 (94.9%)^a^
	Coverage of assessments	8 + signs–91.9% 9 + signs–78.8%	2116 (75.7%)^b^
	Referrals made	101 (13.1%)	279 (10.0%)

^a^This represents weight assessed at first postnatal visit.

^b^This represents babies who have had a full assessment for all the 10 signs.

### Conceptual framework for the evaluation of assessment and referral component

**Figure 2.**
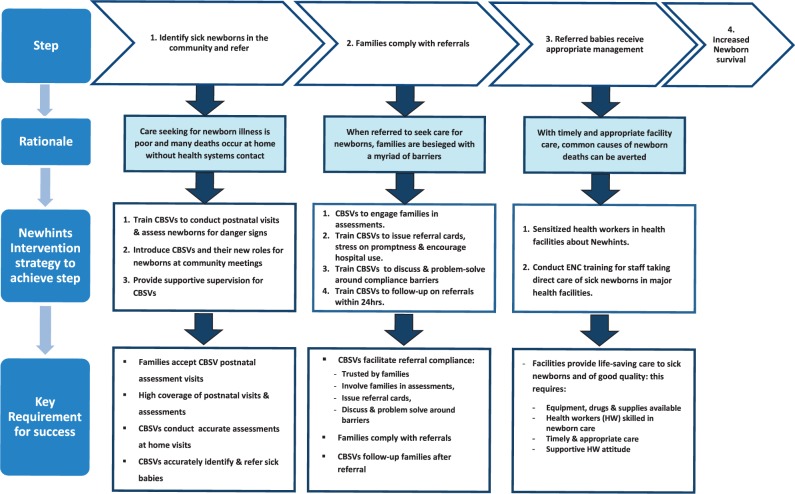
Conceptual framework for increasing access to care for sick newborns through community volunteer assessment and referral.

Figure 2 shows the conceptual framework adopted by the Newhints intervention for increasing access to care for sick newborns through community assessment and referral as a strategy to improve survival. There are three main steps, each with a specific goal. These are (1) sick newborns are identified in the community and referred (2) families comply with referrals and (3) referred babies receive appropriate management at health facilities. The framework shows the rationale for each step, the strategy used to achieve the goal (outlined below) and the key requirements for success. The rationale and the evaluation of the key requirement for success are discussed in detail in the section on findings, drawing together data from the formative research and the process evaluation.

### Step 1

#### CBSV training

CBSV training was in three phases, totalling 9 days. The first phase (3 days, in March 2008) covered behaviour change communication, counselling skills, promotion of ENC practices and saving for emergencies in pregnancy, childbirth and for the newborn. The 4-day second phase in June/July 2008 focused on assessment and referrals. It involved interactive practical newborn assessment video exercises using the WHO Integrated Management of Childhood Illnesses (IMCI) Computerized Adaptation and Training Tool (ICATT) (World Health Organization 2007). One day was dedicated to clinical practice sessions at the major health facilities, where each CBSV trainee assessed at least two babies using digital clinical thermometers, stopwatches and portable weighing scales with colour-coded bands: red for weights below 1.5 kg identifying very low birthweight (vLBW) babies; yellow for weights between 1.5 and 2.4 kg identifying LBW babies; and green for weights of 2.5 kg and above. Decision making around referral, facilitation of referral compliance and problem-solving skills were discussed in detail using case stories and cards with various weights, respiratory rates and temperature measurements.

The third phase was a 2-day refresher course in October 2008 which was convened in response to supervisors’ feedback. It focused solely on the assessment and referral decision-making including additional clinical practice sessions in the major health facilities.

#### Community introduction of CBSVs

A series of activities were carried out within communities to promote awareness about the Newhints intervention and increase acceptability of CBSV visits. These included meetings with community opinion leaders and traditional birth attendants (TBAs) as well as community-wide ceremonies with community members in attendance where certificates were awarded to CBSVs for successful completion of the newborn assessment training.

#### Supervision of CBSVs

CBSVs were supervised by trained district-based project supervisors (DiPS) who visited CBSVs monthly. The DiPS were experienced field supervisors with a minimum of secondary school education who were trained and paid by Newhints but seconded to the DHMTs. There were two DiPS per district and they were each provided with a motorbike for the supervision of an average of 30–35 CBSVs. Their supervision included replenishing CBSV supplies as well as joining them on a repeat home visit and providing supportive supervision, observing and recording their performance on a structured directly observed supervision (DOS) form and providing feedback at the end of the session. The DiPS also organized bi-monthly zonal group sessions for the 8–12 CBSVs within a zone to discuss overarching community concerns and problem-solve around them.

### Step 2

CBSVs actively ‘engaged family members’ who were involved in the care of the newborn during the assessment. When a baby was identified with a danger sign, they issued the family with a ‘referral card’ to take along to the health facility, dialogued with them to elicit barriers to compliance and ‘problem-solved’ around these barriers. They also conducted a ‘follow-up visit’ within 24 h of referral to check compliance and when mothers failed to comply, they re-assessed the baby and referred to a health facility if danger signs persisted. They also dialogued to elicit the barriers and dialogued with families to overcome these.

### Step 3

‘Sensitization sessions’ were organized for all facility care providers in the study area to introduce Newhints and to harmonize Newhints CBSV messages with those of the Ghana Health Services (GHS). Implications of the intervention on GHS routine services and the use of the referral card for identifying referred sick babies were also discussed. Newhints also facilitated a WHO-sponsored 4-day ENC facility training course for staff who took direct care of sick newborns from the top 15 facilities including the four main district hospitals in the study area. These were selected to cover facilities with a minimum of 50 births per year, which the surveillance data confirmed as locations where most births and sick newborn care occurred.

### Evaluation data collection

Data were gathered to evaluate each requirement in the conceptual framework from five sources: process data; supervisory (DOS visit) records; quality control of DiPS assessment; health facility assessment (HFA) and in-depth Interviews including referral narratives (IDIs) with mothers, CBSVs and facility care providers. All data collection tools were paper-based.

#### Process data

Process data were collected from a sub-sample of 4006 recently delivered mothers in the Newhints intervention zones. This comprised 64 mothers randomly selected each week from March to July 2009 from the trial surveillance database and all mothers who delivered between August and December 2009. These data covered CBSV visits, assessments, referrals, compliance, type of health facility used and care provided using pre-tested data collection forms, administered by trained field supervisors.

#### DOS records

DiPS completed records for 759 DOS visits between May and December 2009 in which newborn assessments were observed. Information extracted from these forms included the quality and content of the CBSV assessments, referrals made, advice given and repeat measurements made by the DiPS.

#### Evaluation of the quality of DiPS’s assessment

An evaluation of the reliability of the DiPS assessments was carried out in November 2009 at the four main hospitals by the study clinician (AM) assisted by a research officer. Each DiPS assessed four babies and recorded their findings onto a structured form. These assessments were observed by the study clinician who independently noted down his assessment findings. Both AM and the DiPS handed their forms to the research officer for compilation.

#### Health facility assessment survey

Details of the HFA survey have already been published (Vesel *et al.* 2013). In brief, all 86 health facilities (public and private) serving mothers and babies in the Newhints trial areas were visited between July 2009 and March 2010. Respondents were matrons (in-charge) of the maternity/newborn care units or the facility. The assessment covered: essential infrastructure, availability of equipment, drugs and supplies for newborn care; services provided; and clinical vignettes which depicted clinical case studies of newborns with respondents asked to describe the care that should be provided in these cases. Newborn conditions covered included resuscitation, thermal care, feeding practices, care of vLBW babies and discharge procedures.

#### In-depth interviews

IDIs were conducted between June 2009 and March 2010 with three groups of respondents using saturation sampling with the sample size determined by conducting interviews until no new information arose. IDIs lasted between 45 and 90 minutes and were digitally recorded. Fieldnotes on the setting, perception of the mothers’ socioeconomic status and nuances that added context to the responses were taken.

Fifty-five recently delivered mothers with babies referred by CBSVs were selected from the process database using purposive sampling to obtain balance on age, educational attainment, marital status, residence, ethnicity, parity and compliance with referrals. IDIs involved a narrative of the referral experience complemented by probing using a pre-tested interview guide to cover details of experiences from the CBSV assessment, referral, compliance decision making, compliance, facility used and care provided, outcome for the baby, and CBSV follow-up visits.

Similar IDIs were also conducted with 21 CBSVs who had referred babies, purposively selected from the trial CBSV database to cover all ages, level of education, gender and district. Topics covered in these IDIs included the number of babies they had referred, a detailed narrative of the most complicated referral, family reactions to the visits and the referrals, their perceptions on barriers and facilitating factors to families compliance, care provided to referred babies as reported by families, and their experiences at the follow-up visits.

IDIs were also conducted with 15 facility care providers covering all levels of staff that mothers would come into contact with including a paediatrician, doctors, nurses, midwives and front-desk staff. The interview covered experiences with Newhints referred babies and their mothers, perceptions on the validity of the CBSV referrals, mothers’ expectations of care, care provided for newborns, and challenges with providing this care.

### Data analysis

Data analyses were carried out in Stata version 11.2. Principal components analysis was used to calculate an asset index (using household assets) from which socioeconomic quintiles (SEQs) were derived after ranking mothers and dividing them into quintiles. Agreements between assessments were compared using Kappa statistics, with the DiPS as standard for the DOS assessments and the clinician for the DiPS’s evaluation. The interpretation of the Kappa was based on acceptable standard (Viera and Garrett 2005) where 1 means perfect agreement and 0 means no agreement. Kappa of <0.40 was interpreted as fair or slight agreement, 0.40–0.60 moderate agreement, 0.61–0.80 as substantial agreement and 0.81–0.99 almost perfect agreement. Sensitivity and specificity of CBSV assessments and referrals were also estimated and 95% confidence intervals (95% CI) were reported on all estimates.

The IDIs were transcribed into MicrosoftWord by combining the recordings with the fieldnotes. Analyses were done in NVIVO 9.2 and involved generation of themes from multiple reading of the transcripts, systematic indexing/coding of the data into these themes and exploration of relationships and their contextual interpretations.

### Ethical considerations

All respondents for the interviews provided individual informed consent for the interviews after the rationale for the study and the benefits were explained to them. Respondents were assured of confidentiality of their responses and their right to decide to participate or not without any effect on the care they received at facilities. They were told they were also free to withdraw from the study at any point. Consent was indicated with a signature or a thumbprint. Newhints and this evaluation received ethical approvals from London School of Hygiene & Tropical Medicine (LSHTM) and Kintampo Health Research Centre (KHRC). Newhints is registered at clinicaltrials.gov (Number = NCT00623337).

### Role of the funding source

The Newhints Home Visits CRT was funded by the World Health Organization, Save the Children's Saving Newborn Lives programme, from The Bill & Melinda Gates Foundation and United Kingdom Department for International Development. Funders had no role in data collection, data analysis, or writing of the manuscript. The corresponding author had full access to all data and, together with the last author, the final responsibility to submit for publication.

## Results

### Step 1: Identify sick newborns in the community and refer

#### Rationale

The rationale for this step was that formative research leading to the implementation of Newhints found that families do not recognize illnesses in their newborns within the homes and care seeking for sick newborns is poor (Bazzano *et al.* 2008; Okyere *et al.* 2010). IDIs with mothers and CBSVs confirmed the need for this approach. The majority of families had not recognized their newborn was ill before the CBSV’s assessment. Also recognition without action happened.

*“At times it can be very difficult because the family members do not know that the baby is sick but because I have already discussed things with them at the pregnancy visits, they learn to trust me and so they comply.”* (27-year-old female CBSV, a teacher by profession)

*“I saw that the baby was discharging from the eyes and there were rashes on the body but I did not do anything about it. As for the breathing, I have never seen babies breathe before and so I did not know until he came. And the hot body too, I thought that was the way newborn babies were and so I did not think it was any problem.”* [24-year-old Dagarti primip, junior high school (JHS) graduate]

#### Acceptability of assessment visits

Both mothers and CBSVs reported that the Newhints assessment visits were welcomed and acceptable to families. Mothers were happy that the work of the CBSV was helping them know when their newborns were ill to seek care. Some explained that they were pleased with the assessment visits because it was reassuring to know the state of health of their newborns.

*“The way he has the patience to visit us three times to check the health of the baby is very good. Sometimes your baby might be sick but you may not know so if he comes to do this work to check whether baby has a ‘problem’ and tells you to go to the hospital, it is really good and it helps we the mothers; when he says there is no ‘mistake’ you the mother also feels free.”* (38-year-old Bono farmer)

The CBSVs also confirmed that they were well received and that other family members who were invited to participate in the assessment joined in the discussions around the findings. They added that families were in fear their newborns could die if the babies had an illness and they did not know and therefore positively demanded assessment visits. The demand was reinforced by hearing experiences from other mothers whose babies had been referred and successfully treated at the facility.

*“They really understand the work I am doing so most of them invite me to come for the assessment. It seems they see the benefits that those who allow me to examine their babies get and so they too wanted to have that.”* (49-year-old female Bono CBSV)

#### Coverage of CBSV assessments and referrals

Table 1 shows details of the CBSV assessment and the percentage of assessments during which they checked each of the danger signs. The latter is based on the DOS forms completed by DiPS during supervisory visits and on reports from mothers in the process sample. Process data showed that 70% of mothers received CBSV visits in the postnatal period, and that at these visits, 76% of babies had their respiratory rates counted, temperature taken and weights measured. Coverage of these assessments individually was very high, ∼95% on each. DOS data confirmed this high coverage of both individual and complete assessments. CBSVs were observed to check for at least eight danger signs in 92% of visits, and for 9 or 10 danger signs in 79% of visits. The DOS data also shows that, on average, 95% of the assessments that required the use of instruments were conducted as compared to 88% of those checked by observation. Thirteen per cent of babies had danger signs and were referred at DOS visits compared with 10% reported on the process form.

#### Accuracy of CBSV assessments and referrals

Table 2 shows that CBSV assessments strongly agreed with the DiPS assessments made during the DOS visits; with coefficients of agreement between the two ranging between 0.75 for count of respiratory rates and 1.0 for lethargy (or when baby moves only when stimulated) or vLBW babies, indicating excellent to near perfect agreement. Apart from observing for local infections, the sensitivities of CBSVs diagnosis for signs checked by observation were relatively low (57–59%) with just >40% detected by the DiPS missed by the CBSV; the exception was local infections with a sensitivity of 95%. The sensitivity was also high for danger signs using instruments (80–100%). However, specificities were close to 100% for all danger signs, except for the confirmatory second respiratory rate count that had a specificity of 91%. The evaluation of the DiPS quality of assessment also showed that the DiPS achieved near perfect agreement with the study physician; Kappa = 0.9–1.0. These findings suggest that CBSVs can accurately assess babies for danger signs at home visits.

**Table 2.** Accuracy of CBSV assessments compared to their supervisors (the District-based project supervisors (DiPS) during directly observed supervisory (DOS) visits (*N* = 759)

Danger sign	Danger sign present (based on DiPS assessment)^a^	Agreement (%)	Kappa (95% CI)^b^	Sensitivity (95% CI)	Specificity (95% CI)
Observed sign					
Chest in-drawing	22 (2.9%)	99.3	0.85 (0.71, 1.00)	59.1% (36.4%, 79.3%)	99.9% (99.3%, 100.0%)
Only moves when stimulated	7 (0.9%)	100.0	1.00 (1.00, 1.00)	57.1% (18.4%, 90.1%)	100.0% (99.5%, 100.0%)
Yellow soles	14 (1.8%)	99.6	0.84 (0.66, 1.00)	57.1% (28.9%, 82.3%)	100.0% (99.5%, 100.0%)
Local infections (Eye/skin/cord)	61 (8.0%)	99.6	0.97 (0.94, 1.00)	95.1% (86.3%, 99.0%)	100.0% (99.5%, 100.0%)
Measured with instrument					
Respiratory rate (first count) 60+/minutes	93 (12.3%)	94.9	0.75 (0.67, 0.83)	73.1% (62.9%, 81.8%)	97.5% (95.9%, 98.5%)
Respiratory rate (second count) 60+/minutes	57 (7.5%)	91.6	0.83 (0.69, 0.96)	92.7% (80.1%, 98.5%)	91.2% (76.3%, 98.1%)
Hypothermia: temperature <35.5°C)	10 (1.3%)	99.9	0.94 (0.82, 1.00)	80.0% (44.4%, 97.5%)	99.9% (99.3%, 100.0%)
Fever: temperature >37.4°C	23(3.0%)	99.3	0.90 (0.81, 0.99)	100.0% (85.2%, 100.0%)	99.3% (98.4%, 99.8%)
vLBW (<1.5 kg)	1 (0.1%)	100.0	1.00 (1.00, 1.00)	100.0% (2.5%, 100.0%)	100.0% (99.5%, 100.0%)
**REFERRED**	**127 (16.7%)**	**96.6**	**0.87 (0.82, 0.92)**	**79.5% (71.5%, 86.2%)**	**100.0% (99.4%, 100.0%)**

^a^The column labelled ‘danger sign present based on DiPS assessment’ represents the proportion of the newborns assessed who had a particular danger sign.

^b^Kappa is the statistical coefficient of agreement between the DiPS and the volunteer. A high Kappa means high agreement and conversely a low Kappa means poor agreement; *P* < 0.001 for all the Kappa statistics.

#### Accuracy of referrals

Referral decisions made by the CBSVs at these DOS visits also achieved excellent agreement with the DiPS; Kappa = 0.87 (0.82, 0.92), with 80% sensitivity and 100% specificity. CBSVs are accurately referring babies based on the danger signs they noted with no false positives but failing to refer some as they had failed to detect some signs. Validity and accuracy of CBSV referrals also emerged as a theme in the IDIs with facility care providers. They commended the diagnostic acumen of the CBSVs and confirmed that the majority of their referrals were valid and accurate.

*“they sometimes identify problems that even some of us struggle to find; I think whatever training they were given must have been of a very good standard*.’ (a medical doctor in a district hospital)

### Step 2: Families comply with referrals

Formative research identified that mothers’ ability to seek care for sick newborns was often besieged with many barriers including costs, distance to facilities and norms and beliefs that some illnesses such as a culturally constructed syndrome of ‘Asram’ (Okyere *et al.* 2010) were ‘not-for-hospital illnesses’ so that, even when these illnesses were identified, appropriate care was not sought (Hill *et al.* 2003; Bazzano *et al.* 2008; Okyere *et al.* 2010). Addressing these barriers was seen as key to achieving high compliance with referrals. The Newhints strategy therefore explicitly did so by training the CBSV to engage families during the assessments and involve them in the decision making around the referral. They were also trained to issue referral cards to the mothers whenever a baby was referred, to stress the importance of promptness of compliance, and to encourage them to take the baby to a hospital. They then elicited any barriers that the families were facing in being able to take the baby to the hospital and problem-solved around them. The CBSVs returned the next day for a follow-up visit to check compliance. If the baby had not been taken to a health facility, they re-assessed and referred again if the danger signs persisted. For these mothers who could not comply, they also enquired to know what the barriers were and supported them to overcome these barriers. This support included soliciting funds for those who did not have means of transport, providing further explanations on newborn vulnerability or involving other decision makers in the discussions and soliciting their support for the mother to go. In a few instances, they called their supervisors (the DiPS) to intervene and families then perceive the seriousness and complied.

### CBSVs facilitate referral compliance

#### Trust for CBSVs

Trust by families was seen by CBSVs as crucial to convincing mothers to comply with referrals. In their IDIs, CBSVs thought families trusted them because of their enhanced profiles as ‘doctors’ for their communities and were cautious to protect this reputation by promptly referring babies to facilities. They attributed the high acceptability of the visits to the use of instruments such as thermometers and respiratory counters for the assessments. CBSVs mentioned that when families saw the instruments they were using to assess their babies, the families were convinced that CBSVs were knowledgeable. This perception further motivated the CBSVs and made them assume responsibility for the health of babies within their communities. They perceived that if they CBSVs failed to refer a baby and the baby dies, they will be seen as incompetent.

*“We know she is a doctor and knows her job so we decided to listen to her advice. We were ready to send the baby and this decision was easy for us because she is a doctor.”* (20-year Mo mother with 8-year formal education)

*“If I see a newborn and do not refer and something happens, they will carry the news around town that even a doctor came to see the baby but did not know that the baby was sick and that is why the baby died. If I refer them, I know the baby will get well and I will also have my peace of mind”* (46-year-old male Bono CBSV; father of seven)

#### Involved families in assessments

DOS data showed that 84% of the times, CBSVs involved family members, other than the mother, in the assessment and the discussions of the findings. In their IDIs, mothers, other family members and the CBSVs, confirmed involvement of other family members in discussions around referrals and compliance:

*“I entered the room with him where the baby was and when we got there, he (CBSV) said he was coming out again to wash his hands. He came out and washed his hands and asked me to call everybody at home who normally helped in the care of the baby. At the time, my mother and my eldest daughter were around and so I called them to join us.”* (38-year Mo mother of five with 3 years of formal education)

“*When I got to the house, I invited ‘the man of the house’ to come and participate in the visit. During the pregnancy whenever I invited him, he always said I should go ahead and have the meeting with the women. On that day, the baby was crying excessively and so when I invited him for the assessment he got interested and came to sit to see what I did.”* (48-year-old CBSV; Baby was referred and husband accompanied the mother and baby to a hospital.)

#### Issued referral card

During the DOS visits, CBSVs issued all mothers whose babies were referred with referral cards. In their narratives, 73% of the mothers suggested that with the referral card, they thought the baby’s illness was severe and moreover the CBSVs explained to them that with the card, they were going to be seen promptly at health facilities. CBSVs also confirmed this adding that the card made mothers want to go. When describing how they identified Newhints babies, facility care providers mentioned that they always came bearing the referral card. They added that, with the card, mothers wanted to be treated quickly even if they came to meet other people in the facility waiting to be attended:

*“He gave me a card, it was a yellow card and said I should take along and if I put it in the hands of the ‘doctors’, it will make them see the baby quickly for us.”* (24-year-old Bono mother of two)

*“I tell them not to join the queue but to go directly to the nurses and tell them that they were from Newhints with showing of the yellow card and they will be taken care of and that makes them go.”* (21-year-old CBSV)

*“The mothers come with a card. They have a special card that they give to them to bring along. At times when you ask the mother, she says ‘a boy came to check my baby and asked us to come and see the doctor. When you look at the card, you see they are from Newhints.”* (46 years enrolled midwife)

*“You will see that yellow card, and then they want to be treated quickly; even though they come to meet other people here they want to be treated early.”* (57 years senior midwifery officer)

#### Overcoming barriers

The CBSVs elicited perceptions of vulnerability around newborns in the families to emphasize the need for prompt compliance with referrals. Other barriers such as cost and distance ceased to be important considerations once the baby’s illness was perceived to be severe. This removal of compliance barriers was also related to emergency preparation during pregnancy; data showed 86% of mothers said they saved during the pregnancy for emergencies and 87% also enrolled on the National Health Insurance Scheme which provided free facility care for sick newborns.

*“I could then see clearly that the child was very sick after he explained to us so I was ready to send him to the hospital.”* (15 years Bono mother with 7-year formal education)

*“‘he told us to go to the hospital the same day; he came to the house at around 8-9 in the morning but I explained that my mother was not around at the time because she had gone to the farm. I could not carry the baby by myself to the hospital because it was my first delivery and I did not have the experience.”* (20 years primip; a teacher)

*“at the time he was visiting us in the pregnancy, he told us to save some money in the form of ‘susu’ so that when we are going to deliver or if we get an emergency, we could use for the costs and we did.”* (35-year mother; a farmer)

In some cases, when mothers were found to be handicapped and could not afford to take the baby, CBSVs contacted other family members to solicit support to enable the mother to comply with the referral. They also directly and personally supported mothers with loans and gift money to enable them to comply although the project did not provide funds for this and the token five dollars ($5) per month paid to them by the project was just to motivate them. Where mothers thought transport was the barrier, CBSV went to get a vehicle for them or negotiated for them to be given the priority to take their sick baby to hospital:

*“After telling us, the CBSV accompanied me to my husband’s house to disclose his findings to him and his brother (they live in the same house). There, immediately he finished, the man (husband) did not even ask any question and just went and brought me money to take along to the hospital. They believe him ‘very much’.”*(18-year-old Dagarti farmer and primip)

*“I told him that I would wait and go the next morning but he said he wanted me to go the same day. He then offered to go to the roadside and see whether he could get a vehicle for me to take to the hospital and Nsawkaw but when he went and did not get one, he came back to inform me but still wanted me to go and so I rather walked to Seikwa.”* (23-year-old Sisala primip, completed JHS)

#### Referral compliance

Process data showed that compliance with referrals was unprecedentedly high with 86.0% (95% CI = 81.4–89.9%) of mothers taking their babies to a health facility, three-quarters of these going to hospitals (A Manu, Z Hill, G ten Asbroek, S Soremekun, T Gyan, B Weobong, submitted for publication). There was evidence to suggest that compliance was pro-poor with the poorest mothers complying more than the least poor (88.4 vs 69.7%; *P* = 0.003) and rural residents more than urban (87.3 vs 81.7%; *P* = 0.02) (A Manu, Z Hill, G ten Asbroek, S Soremekun, T Gyan, B Weobong, submitted for publication) Although distance did not seem to affect compliance, given the spatial spread of referrals and mothers who complied with them showing no evidence of clustering (Figure 1), urban mothers who lived closer to the hospitals had better means of transport and were able to reach facilities faster than rural ones.

#### Follow-up visits

The DOS data showed that CBSVs assured families that they were going to return for follow-up visits in 92% of all the referrals they made. In IDIs with the mothers and the CBSVs, they indicated that this assurance to return and check on compliance made mothers want to comply. CBSVs were also motivated to follow up on referrals because they wanted to know what happened in the facility; the mothers appreciated this.

*“He gave me a card and said he would come back later to check if I have been able to go. What am I going to tell him if he comes and asks and I have not been able to go?”* (40-year Bono mother of eight)

*“Yes, I think so! If I had not told them I will return to check the next day, even if they would have gone, they would not have gone on the same day—they would have waited for some time before taking action.”* (39-year male Mo CBSV**)**

### Step 3: Referred babies receive appropriate management

The rationale for this step was that timely and appropriate management of sick newborns can prevent newborn deaths (Darmstadt *et al.* 2005; Qazi and Stoll 2009). Our formative research showed that even though hospitals in the study area were capable of managing sick newborns because they have the equipment, drugs and infrastructure, technical skills of staff were lacking (Howe *et al.* 2011). The Newhints team therefore organized the facility ENC training for staff in the largest facilities. No other direct intervention (such as supply of drugs, equipment or changes in infrastructure) was made within the health facilities.

#### Equipment, drugs and supplies

The health facility assessment survey (Vesel *et al.* 2013) showed that only hospitals had all the requisite equipment, drugs and supplies for the management of sick newborns. However, even though these hospitals were connected to the national power grid, the power supply was not reliable and only two had stand-by generators. There was over-reliance on equipment such as incubators which were inadequate in number. These incubators usually carried more than two babies at a time. Some of these were sick babies whilst others might not be sick but vulnerable such as LBW babies. The risks of nosocomial cross-infection were very high. Only one had a dedicated newborn care unit. Kangaroo Mother Care for premature or LBW babies was not practised.

#### Health worker newborn care skills

Newhints ENC training did not seem to make any lasting difference to the quality of newborn care provided in the trial districts. Apart from one paediatrician, no health worker had had specialised/formal training in newborn care. Doctors and clinicians failed to attend the Newhints facility ENC training. Instead nurses and midwives who did not provide definitive treatment for newborns attended. The health facility assessment found that only 19% of nurses or midwives reported as capable of managing sick newborns were at post in the top eleven health facilities (Vesel *et al.* 2013) and these were mainly the respondents to the assessment questionnaire. Just over 10% of these had been trained in facility ENC. Follow-on interviews revealed that staff placement policies played a role in the skills deficit; some ENC-trained staff who were still working in the same facility had been moved to other units where their newborn skills were not utilized; others had left the facility altogether. Moreover, management protocols for sick newborn care were non-existent in all the facilities.

*“…but the other is the question of quality and quality; because even for the older nurses, with no additional training, they cannot do what you expect them to. When the experienced few are on leave, it leaves you with nobody to step in.”* **(**a paediatrician)

*“There is none; we keep our protocols in our heads and teach the juniors among us how we work here.”* (a senior midwife)

There were suggestions, however, from care provider responses in the IDIs that if trained staff were placed properly and supported, the outcome for sick newborns could have been different. Respondents who had additional training in sick newborn care seemed to have better understanding of newborn vulnerability and had a different attitude towards Newhints referred babies:

*“…as for newborns, their conditions can change very quickly and if I let them go, I do not know what next will happen and so I will not take the chance.” (*A midwife trained by the paediatrician to support in a newborn care unit)

*“Mostly they say the baby is having fast breathing. Some are due to cord sepsis. I think if infection is setting in, fast breathing is the first sign. So when you see fast breathing and you send them home, you might be doing the wrong thing. I detain them overnight and oftentimes, sepsis is seen by the next day. In some cases you see reddening around the cord so the doctor then puts them on five days of antibiotics.”* (an ENC-trained midwife)

#### Timely and appropriate care

Table 3 shows evidence of substantial delays within health facilities before sick newborns were seen. These delays were worst in the four main district hospitals where over a third of mothers were kept waiting for more than three hours. These delays sometimes resulted in deaths. Also, Newhints process data showed that about a quarter of referred babies were sent home without treatment often with the decision made without proper examination of the newborn (A Manu, Z Hill, G ten Asbroek, S Soremekun, T Gyan, B Weobong, submitted for publication). IDIs with mothers, CBSVs and doctors confirmed that some babies subsequently died after health facility contacts:

*“I referred the baby in the morning at around seven o’clock. The mother said she took the baby to the hospital and the nurse there didn’t attend to her… She said the nurse was angered by her home delivery saying ‘if you sit at home to deliver and there is a problem, then you are rushing over to us!’ The nurse directed her to wait and see the doctor but the baby died before the doctor came.”* (47-year-old CBSV)

**Table 3.** Timeliness of care at health facilities for mothers who complied with referrals

Waiting time before first health worker contact	Type of health facility: *n* (%)		
	Four main district hospitals	Other facilities	Total
Less than 30 min	25 (15.5%)	30 (38.0%)	55 (23.7%)
30+ minutes but less than 1 h	37 (23.0%)	20 (25.3%)	57 (24.6%)
1 h but less than 3 h	41 (25.5%)	15 (19.0%)	56 (24.1%)
3+ hours	55 (34.2%)	9 (11.4%)	64 (27.6%)
Total	158 (68.1%)	74 (31.9%)	232^a^ (100.0%)

^a^Details were missing for eight respondents.

*“we have nothing to say about how they treated us over there ‘bro’ (interviewer)…they are doing their work and they said there was nothing wrong with the baby but he died, what can you do?”* (35-year-old Sisala mother who lost her 2nd twin after she complied with referral and was sent home without treatment)

*“I think because of the workload, pressure and human resource constraints, there’s usually not much time to spend evaluating babies; and so newborns that could otherwise be unwell can be just glossed over and think that they can go home, send them home and they deteriorate and pass away.”* (a medical doctor)

#### Supportive health worker attitudes

Staff attitudes were perceived as very poor with both CBSVs and mothers suggesting that interventions to improve families’ experiences within facilities should be a priority for continued or future implementation of the Newhints intervention. Mothers reported being abused when they took their sick newborns for care in the facilities especially if they delivered at home or failed to attend ANC during the pregnancy.

*“When I got there, she asked what was wrong with my baby and so I showed her the yellow card. There and then, she got so angry and threw the card at me and threw me out because I delivered at home.”* (35-year-old mother of four)

*“Mostly, the women (nurses) shouted at and manhandled her and I told them she’s never given birth before. They said she shouldn’t stay inside the room whilst they treated the baby. Even if the baby cried they didn’t allow her see to him.”* **(**a grandmother of 15-year-old first-time mother)

## Discussion

A summary of the key lessons learned, the strength and weaknesses of the evaluation, how the evidence generated compares with prevailing knowledge about CHW assessment and referrals, and overall conclusions are presented in the next four sections.

### Summary of lessons learned

1. Family recognition of sick newborns remains very poor and recognition without action is common. Home visits to identify and refer sick newborns are a necessary and effective strategy to improve access to care for sick newborns. These visits are welcomed by families.
2. Training CBSVs to conduct home visits and accurately assess and refer sick newborns can be achieved in just 9 days. Six of the 9 days focused on this component with 2 days of clinical practice sessions. Scale up should therefore be logistically feasible to achieve, even in LMIC settings with weak health systems that may not afford to have staff away on training for long periods.
3. The use of the clinical practice sessions are crucial to build volunteer confidence at handling newborn babies. It provides practical exposure to newborn assessments as will be encountered within communities and the opportunity to interact with mothers, most of who hail from communities comparable to those of the CBSVs.
4. A simple checklist for danger signs with referral when any one of them is present works well with community volunteers, and is preferable to a clinical type algorithm. The checklist approach takes less time to explain, is more easily understood and does not appear to lead to false positive referrals.
5. Effective supervision and monitoring is essential, and should include observation of home visits to reinforce skills and ensure and maintain quality implementation of this strategy. These observations can be best achieved by carrying out additional visits to newborns rather than relying on supervision coinciding with scheduled home visits, as these do not happen on a regular basis.
6. Supervised home visits had the unexpected benefit of enhancing the volunteer profile in the community and associating them with the health services, reinforcing the importance of compliance with any referrals.
7. With proper facilitation and planning, high compliance with CHW referrals is achievable even for rural families. However, distance to referral level facilities remains a barrier in ensuring prompt access to care for sick newborns.
8. Increasing access to care through community assessment and referral is a pro poor approach with the potential to reach all newborns regardless of wealth or place of residence, as confirmed by the high compliance rates achieved across socioeconomic quintiles and in rural as well as urban areas.
9. Issuing a referral card could make a difference. It has several roles. It emphasises the importance of the referral, promotes a sense of continuity between community volunteers’ assessment and referral and facility care, and allows effective triaging of referred newborns at health facilities. All these were achieved with the use of the Newhints referral card except that triaging was not effective because, due to poor quality of care in facility, treatment was delayed and resulted in preventable mortalities.
10. Increasing access to care for sick newborns is necessary but not sufficient to ensure newborn survival; it must be matched with improved quality of facility care. This should be tackled in parallel to implementation of home visit programmes not only through health worker training, but through on going quality improvement strategies.
11. Community-based assessment and referrals could lead to increases in workload at health facilities especially which impact on the quality of care and should be an early consideration in implementation. However, if CHW assessment and referrals have high specificity, as was the case in Newhints, increased facility workload is probably indicative of the unmet need for newborn care within communities.
12. Community-based strategies that increase access to care for sick newborns may not be perfect; there is always the possibility of false positive referrals. However, these may have merits in that they provide ‘opportunistic’ contacts with families who were otherwise not reachable within routine health programmes. In addition, encouraging such referrals will likely result in sick newborns being seen early which may prove economically and medically prudent—reducing facility expenditure per capita sick newborn and result in better outcomes.
13. With the proven ability of CBSVs to accurately assess newborns and in many instances detect danger signs of illness, a possible modification might be that they are also trained to treat minor ailments in the home and provide pre-referral antibiotics in recognition of the long distances to facilities. However, caution needs to be exercised as this may inadvertently reduce referral compliance. This unexpected consequence may explain the difference in the very high compliance achieved in Newhints which did not include any treatment, and the much lower compliance observed in the other trials that did. These compliance rates are sustainable because they were not based on provision of resources to the families but rather on helping them identify newborn illness, understand newborn vulnerability and make an informed decision on seeking care. Motivation of the volunteer including effective supervision were also key but if quality of care including families’ experiences at facilities improve, compliance may improve further.

### Strengths and limitations

This evaluation followed a detailed conceptual framework and covered every aspect of the implementation of the assessment and referral component of the Newhints strategy and its rationale. These details and the lessons learned will provide important information to programme implementers about all aspects of the intervention strategy that need consideration before implementation.

A potential limitation of the evaluation is that the DOS visits measured the ability of CBSVs to conduct the assessments but not necessarily what they did. CBSVs might modify their behaviours because they knew they were being observed. However, process data and the IDIs confirmed that the CBSVs routinely carried out the assessments. Another possible limitation is that the IDIs were conducted by the lead author who was actively involved in the training and implementation of the study. It is possible that responses from CBSVs and health professionals could have been biased. However, all the various sources of data including the IDIs provided a convergent evidence of the success of the implementation. The effect of bias, if any, is therefore likely to have been minimal. Finally, as implementation takes time to bed in, it would have been ideal both to evaluate the impact and the implementation over a longer period.

### Comparison with other evidence

Table 4 compares the Newhints approach to increasing access to care for sick newborns with that used in other trials evaluating the home visits strategy. As can be seen, it is the first trial in sub-Saharan Africa that implemented a community-based strategy to increase newborn access to care through home visits. This was done in close collaboration with DHMTs using an existing cadre of community volunteers (CBSVs) within a programme setting (Kirkwood *et al.* 2010). It is also clear from the table that the short duration of training in Newhints is only comparable with implementation of IMNCI in India in Bhandari *et al*.’s trial which trained for 8 days (Bhandari *et al.* 2012). Most other trials involved training over extended periods of time (Bang *et al.* 2005a; Baqui *et al.* 2008; Kumar *et al.* 2008; Darmstadt *et al.* 2010; Bhutta *et al.* 2011). In many LMICs, the added costs due to provision of training logistics including travel costs for trainees and/or their housing, hiring of venue and compensation for trainers’ times will escalate the cost of implementation. Newhints assessment and referral only draws parity with the Bhandari *et al.* (2012) in the number of postnatal visits conducted by CHWs; all other trials except Kumar *et al.* visited more often in the neonatal period. Kumar *et al.* however did not implement assessment and referral except the use of Thermospots for hypothermia detection (Kumar *et al.* 2008). All but one of the trainers in Newhints were non-clinicians (Kirkwood *et al.* 2010, 2013).

**Table 4.** Newhints assessment and referral of sick newborns: comparison with other trials using CHW home visits

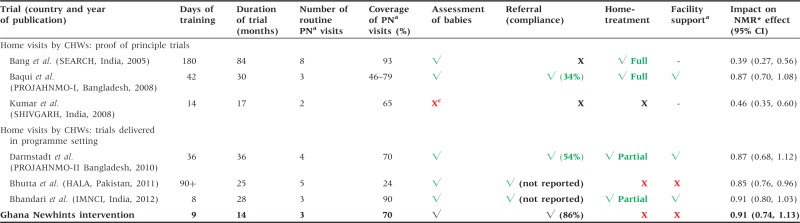

^a^PN postnatal.

^b^Facility support direct intervention in health facilities excluding training such as provision of (or ensuring) drugs, equipment supply, infrastructure etc.

^c^One arm checked for hypothermia; Full implementation includes administration of injectable antibiotics, Partial minus injectable antibiotics. *Neonatal mortality rate; **Project for Advancing the Health of Newborns and Mothers.

Notably, of all the trials that implemented the home visits strategy, Newhints was evaluated over the shortest duration of implementation (14 months) but the results show that coverage of postnatal visits in Newhints compares with many other trials that were implemented for longer (Table 4). There was a progressive increase in the coverage of the intervention driven by revisions in the implementation strategy to make the supervision more effective (Kirkwood *et al.* 2010).

The unprecedented high compliance with Newhints referral is the most important finding of this evaluation (A Manu, Z Hill, G ten Asbroek, S Soremekun, T Gyan, B Weobong, submitted for publication). No trials have reported such high compliance levels to community volunteer referrals. The checklist for referrals was simple to teach and reliable, drawing heavily from previous Asian studies (Bang *et al.* 1999; Baqui *et al.* 2008; Darmstadt *et al.* 2010) and the WHO multi-country Young Infants Study (Young Infants Clinical Signs Study 2008). Although suggestions from facility care providers may be true that some newborns were wrongly referred to them leading to an increase in their workload, questions still remain about babies sent home from facilities without treatment who subsequently died (A Manu, Z Hill, G ten Asbroek, S Soremekun, T Gyan, B Weobong, submitted for publication; AA Manu, Z Hill, C Tawiah-Agyemang, GT Asbroek, S Soremekun, E Okyere, submitted for publication). The Newhints assessment and referrals achieved very high specificity for CBSV referrals suggesting that the increased facility workload (AA Manu, Z Hill, C Tawiah-Agyemang, GT Asbroek, S Soremekun, E Okyere, submitted for publication) may rather be reflecting the unmet need for sick newborn care within communities.

Facility quality of care is the crucial link between referred sick newborns and survival. This lesson supports the Lancet series’ recommendation that isolated community or facility interventions without linkages between them will not deliver optimal results (Darmstadt *et al.* 2005). Facilities in the Newhints study were ill prepared to provide appropriate management for sick newborns (Vesel *et al.* 2013), similar to findings reported by Opondo *et al.* in another study in Africa. Oftentimes, care for sick newborns is equated to sophistication and high technology but this is erroneous (Darmstadt *et al.* 2005). The other option is to explore the possibility of administering some treatment within communities for minor ailments. CHWs have been trained in Asian studies to administer antibiotics successfully within communities (Bang *et al.* 1999, 2005b; Baqui *et al.* 2008). Whilst this has merits in providing timely and life-saving care closer to the community and could reduce workload at health facilities and its consequent impact on quality of care, it may also have several drawbacks. First it may inadvertently reduce referral compliance and careseeking. Most studies in Asia that employed treatment as part of the strategy recorded very low care seeking and poor compliance with referrals (Bang *et al.* 2005b; Baqui *et al.* 2008; Bhutta *et al.* 2011). Secondly, providing volunteers with algorithms to selectively treat newborns based on set criteria may require complex algorithms with increased training requirements.

These important findings are generalizable to the Ghanaian context and across other settings in sub-Saharan Africa. In Ghana, CBSVs are integral parts of the health delivery system and exist in every village. Their potential could therefore be harnessed for the delivery such interventions. Similarly, in many sub-Saharan African countries with ever-dwindling or lack of health human resource task-shifting has become imperative. The Newhints model with an added strategy to improve facility quality could contribute significantly to neonatal mortality reductions which are urgently needed in these settings.

## Conclusions

In conclusion, this detailed evaluation has demonstrated successful implementation of the assessment and referral component of the Newhints intervention with achievement of every key requirement in the conceptual framework. This has important implications for the implementation of the home visits strategy in other settings in sub-Saharan Africa: CBAs can be used to deliver home visits, they can identify sick newborns through accurate assessments and refer to health facilities for care, and families will comply when asked. Moreover we have demonstrated that this approach is feasible to implement, can be delivered at scale and is potentially pro-poor even when delivered within health systems of resource-limited country settings. However, the home visits approach cannot attain its full potential in increasing newborn survival, while the current poor quality of care within health facilities remains. This is the crucial and missing link that must be tackled in parallel.

## Ethical issues

Newhints and this evaluation received ethical approvals from LSHTM and KHRC. Newhints is registered at clinicaltrials.gov (Number = NCT00623337).
